# Adoption of Electronic Health Records (EHRs) in China During the Past 10 Years: Consecutive Survey Data Analysis and Comparison of Sino-American Challenges and Experiences

**DOI:** 10.2196/24813

**Published:** 2021-02-18

**Authors:** Jun Liang, Ying Li, Zhongan Zhang, Dongxia Shen, Jie Xu, Xu Zheng, Tong Wang, Buzhou Tang, Jianbo Lei, Jiajie Zhang

**Affiliations:** 1 IT Center, Second Affiliated Hospital School of Medicine Zhejiang University Hangzhou China; 2 Department of Burns and Plastic Surgery The Affiliated Hospital of Southwest Medical University Luzhou China; 3 Performance Management Department Qingdao Central Hospital Qingdao China; 4 Editorial Department, Journal of Practical Oncology Second Affiliated Hospital, School of Medicine Zhejiang University Hangzhou China; 5 Center for Medical Informatics Peking University Third Hospital Beijing China; 6 School of Public Health Jilin University Changchun China; 7 Shenzhen Graduate School Harbin Institute of Technology Shenzhen China; 8 Institute of Medical Technology Health Science Center Peking University Beijing China; 9 School of Medical Informatics and Engineering Southwest Medical University Luzhou China; 10 School of Biomedical Informatics University of Texas Health Sciences Center Houston, TX United States

**Keywords:** medical informatics, health information technologies, electronic health records, hospitals, Sino-American

## Abstract

**Background:**

The adoption rate of electronic health records (EHRs) in hospitals has become a main index to measure digitalization in medicine in each country.

**Objective:**

This study summarizes and shares the experiences with EHR adoption in China and in the United States.

**Methods:**

Using the 2007-2018 annual hospital survey data from the Chinese Health Information Management Association (CHIMA) and the 2008-2017 United States American Hospital Association Information Technology Supplement survey data, we compared the trends in EHR adoption rates in China and the United States. We then used the Bass model to fit these data and to analyze the modes of diffusion of EHRs in these 2 countries. Finally, using the 2007, 2010, and 2014 CHIMA and Healthcare Information and Management Systems Services survey data, we analyzed the major challenges faced by hospitals in China and the United States in developing health information technology.

**Results:**

From 2007 to 2018, the average adoption rates of the sampled hospitals in China increased from 18.6% to 85.3%, compared to the increase from 9.4% to 96% in US hospitals from 2008 to 2017. The annual average adoption rates in Chinese and US hospitals were 6.1% and 9.6%, respectively. However, the annual average number of hospitals adopting EHRs was 1500 in China and 534 in the US, indicating that the former might require more effort. Both countries faced similar major challenges for hospital digitalization.

**Conclusions:**

The adoption rates of hospital EHRs in China and the United States have both increased significantly in the past 10 years. The number of hospitals that adopted EHRs in China exceeded 16,000, which was 3.3 times that of the 4814 nonfederal US hospitals. This faster adoption outcome may have been a benefit of top-level design and government-led policies, particularly the inclusion of EHR adoption as an important indicator for performance evaluation and the appointment of public hospitals.

## Introduction

Electronic health records (EHRs) are the most important component of health information technology (HIT), and their adoption rate in hospitals indicates a country’s level of digitalization in medicine. In the United States, EHRs enable the electronic documentation of providers’ notes, electronic viewing of laboratory and radiology results, and electronic prescribing [1]. The 2009 Health Information Technology for Economic and Clinical Health (HITECH) Act allocated approximately US $3 billion to accelerate the meaningful use of EHRs in US hospitals. Ultimately, the adoption rates in nonfederal hospitals increased from 9.4% in 2008 to 96% in 2017 [2,3]. In Asia, the EHR adoption rate increased from 15.1% in 2010 to 58.1% in 2015 in Korean hospitals [4] and from 21% in 2008 to 53% in 2014 in Japanese public hospitals [5]. In Europe, the usage of EHRs in German hospitals increased from 39.9% in 2007 to 68.4% in 2017 [6]. In China, the “Technical Specifications for Hospital Information Platforms based on EMRs” issued by the National Health Commission in 2015 defines electronic medical records (EMRs, corresponding to hospital EHRs) as complete and detailed clinical information resources that are created, stored, and used electronically by medical institutions and are generated and recorded for citizens in all visits to medical institutions [7]. Since 2015, the Chinese central government has invested over US $3.5 billion in HIT and EHRs and has issued 31 national policies and 134 technical standards covering all aspects of medical care digitalization and the construction of a digital medical security system. Thus, in China, EMRs are legal records created in hospitals and outpatient environments that constitute the data source of EHRs [8]. In the United States, the Promoting Interoperability Programs, led by the Centers for Medicare & Medicaid Services (CMS), do not specifically distinguish between EHRs and EMRs. In this study, the term EHRs refers specifically to the definitions provided by the CMS and China’s National Health Commission.

Funding, policy, social organizations, and other factors, which can all greatly challenge any government, affect in-hospital EHR adoption. The most important factors associated with EHR adoption rates in hospitals are policy support and national standards. In the United States, relevant policies and standards include the HITECH Act [9], CMS Meaningful Use programs [10], and Promoting Interoperability Programs [8]. In China, they include the “46312” strategy [11], EMR Grading Evaluation Standards [12], and Hospital Intelligence Service Grading Evaluation Standards [13]. The second greatest factor affecting EHR promotion in both countries is insufficient financial support for digitalization in medicine [14]. Finally, another main issue is the large gap between the expectations of EHRs from clinical medical staff and their actual clinical performance.

As the world’s largest country in terms of both population and number of hospitals, China has a unique medical system [15], with particular challenges affecting in-hospital EHR adoption. Therefore, the progress and difficulties in EHR adoption in Chinese hospitals are an important reference for other countries. First, through consecutive survey data analysis research, the EHR adoption in Chinese hospitals from 2007 to 2018 and the challenges of HIT innovation were summarized, based on the Chinese Health Information Management Association (CHIMA) Annual Survey—the longest and most authoritative national HIT industry survey in mainland China. Second, with the Bass model, we horizontally compared the EHR adoption rates of China and the United States from 2008 to 2017 and analyzed the challenges faced by the hospitals of these countries based on data taken from the Healthcare Information and Management Systems Services (HIMSS) Annual Surveys of 2007, 2010, and 2014. This study provides an overview and suggestions for further advancement of EHRs in hospitals in both countries, shares these experiences with other countries, and promotes global popularization of HIT.

## Methods

### EHR Definition and Function Reconciliation

Due to the differences in medical systems and traditions, a one-to-one mapping of the functions of EMRs in China and the United States is difficult. Nevertheless, we should clarify the definition and function of EHRs in these 2 countries so that the research results can reflect the closest comparable rates.

As for the United States, the EHR evaluation systems have, mainly, 2 aspects. First, for the governmental aspect, the Office of the National Coordinator for Health Information Technology (ONC) divided EHRs into “basic EHRs” (with or without clinical notes) and “comprehensive” EHRs in 2009. The former focuses on data collection and sharing and only needs to be implemented in one ward, while the latter stresses the clinical process based on the former and requires hospital coverage [2,16] (details in Multimedia Appendix 1). Since 2011, to facilitate the realization of a financial stimulus program, the CMS divided EHRs into 3 stages according to whether they are meaningfully used [17]. Each stage requires core objects and optional menu objects. Second, at the industry level, HIMSS Analytics developed an EMR adoption model (EMRAM) in 2005, including levels 0 to 7 based on “how many departments to use, standardization, sharing in hospital, decision support, sharing outside the hospital” [18].

As for China, the National Health Commission has been promoting the construction of EHRs with various policies and financial support since 2010 and issued the latest requirements on the definition and implementation timeline of EHRs in August 2018 [19]. In this requirement, EHR is divided into levels 0 to 8: levels 0 to 2 (low stage, focusing on the data collection function); levels 3 to 4 (medium stage, focusing on data sharing within or between departments and simple clinical decision making); and levels 5 to 8 (high stage, focusing on clinical intelligent decision making, cross-hospital data sharing, and patient self-service; details in Multimedia Appendix 2).

There is no systematic comparative study of the evaluation systems of China and the United States. Our preliminary comparison study of EHRs in the top 2 tertiary hospitals in Beijing found that the Chinese EHR stage 4 hospitals can accomplish most (7 of 11) meaningfully used tasks in the United States [20]. However, the requirements for some specific functions of EHRs in the 2 countries are inconsistent, which complicates one-to-one matching of the 2 standards. Preliminarily, after comparing the common terms of the 2 standards, we think that Chinese EHR stages 3 and 4 roughly correspond to basic EHRs with notes and comprehensive EHRs, respectively. Unlike US EHR standards, Chinese EHR stage 1 requires Chinese hospitals to use the EHR for billing.

We did not use the data from HIMSS EMRAM, which was used in both countries as the research baseline, mainly because of the serious deviation of the sample distribution. Although about 74% of US hospitals passed HIMSS EMRAM stage 5 or above by the end of 2017, in China, the EMRAM is only a commercial trial project in a small number of hospitals. By June 2019, only 58 hospitals participated in the EMRAM evaluation and met or passed stage 6 [21].

### Data Resources

Data on EHR adoption in Chinese hospitals were obtained from the CHIMA Annual Survey of Hospital Information Systems from 2007 to 2018 [22]. These are the only authoritative, national-level, long-term quantitative data of repeated surveys available on the EHR adoption rates in Chinese hospitals. Every March for a decade, the CHIMA surveyed the application of HIT in mainland China, covering 34 administrative regions. Survey respondents included general hospitals, teaching hospitals, specialty hospitals, traditional Chinese medicine hospitals, and integrated Chinese and Western medicine hospitals. In total, each survey was comprised of 9 parts. This study primarily used data from Parts I, IV, VI, and VIII, assessing respondents’ basic information, information system application and adoption barriers, and data standardization.

Data on EHR adoption in US hospitals from 2008 to 2017 were obtained from data briefs by the ONC [2,3,16] and research by Jha et al [1,23-31]. Data on barriers faced in the information system implementation in US hospitals were obtained primarily from the HIMSS Annual Surveys in 2006, 2007, 2010, and 2014 [32-35].

### Technology Diffusion Model and Bass Modeling

As one of our methods, Bass diffusion modeling was employed for the prediction and characterization of the progress in adoption of EHRs. Diffusion theory is an essential branch of communication theory [36]. The Bass model is widely used in the application and forecasting of new products and technologies [37,38], including many medical-related technologies [39-41]. The Bass model has 9 key assumptions [38,41], which mostly satisfy the scenarios of this study. For example, the market potential of a new product remains temporally constant; geographic boundaries of the social system are unchangeable throughout the diffusion.

Bass modeling has 2 important measures. First, the external influence coefficient, called the “innovation” effect and represented as the *p* coefficient, means the probability of using the product under the influence of public media or other external factors among users who have not used the product. Second, the internal influence coefficient, called the “imitation” effect and expressed as the *q* coefficient, depicts the probability of the same users using the product due to the influence of peers who have already used the product [42]. When *p* is high, the model indicates that the new technology has a rapid diffusion at the beginning of the propagation and that diffusion grows more weakly in the subsequent periods. When *q* is high, the model suggests that the new technology spreads slowly in the beginning, but it accelerates with further popularization and expansion. The Bass model is expressed as:


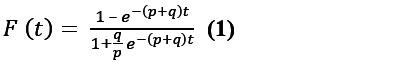


where *F(t)* is the portion of M adopted by time t, *p* is the coefficient of innovation, and *q* is the coefficient of imitation.

### Data Analysis

We conducted statistical analyses and forecasts using linear optimization in Microsoft Excel for Mac 2011 (Microsoft Corporation, Redmond, WA). First, the analyses began with basic descriptive statistics regarding the respondents’ basic information. Second, Bass diffusion modeling was employed to predict the progress of EHR adoption and analyze its characteristics. On one hand, we used the method of least squares to determine the optimal values of *q* and *p*. On the other hand, adjusted R^2^ was used to evaluate the performance of the prediction model. The parameters of the Bass model were trained and estimated using SPSS 20 (IBM Corp, Armonk, NY).

## Results

### Descriptive Analysis

#### Scale and Coverage of the Surveys

**Figure 1:**
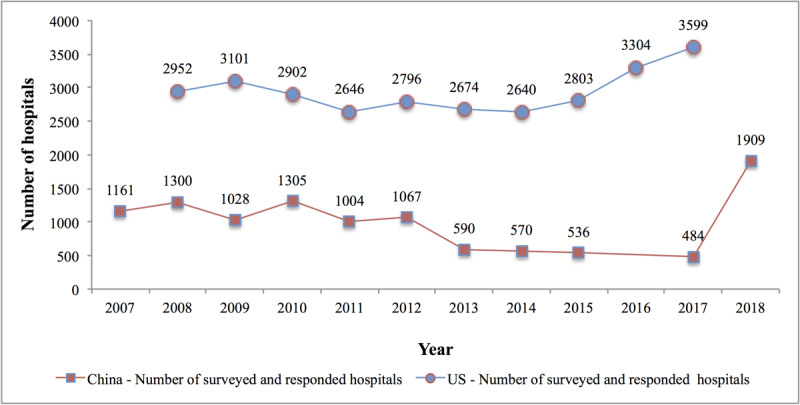
The number of respondents to the 2007-2018 Chinese Health Information Management Association (CHIMA) Annual Surveys of Hospital Information Systems and the surveys on the adoption of electronic health records (EHRs) in US hospitals from 2008 to 2017.

Figure 1 illustrates the number of the 2007-2018 CHIMA Annual Survey respondents (covering over 80% of China’s provinces, municipalities, and autonomous regions) and the number of survey respondents for the adoption of EHRs in US hospitals from 2008 to 2017. In China, all hospitals are classified by the government board into 3 classes: Level I hospitals (roughly equivalent to community-based health centers in the United States), Level II hospitals (county- and municipal-level, small health care facilities), and Level III hospitals (large, advanced general or specialty hospitals) [43]. In this study, hospitals were divided into 2 categories: Level III hospitals vs Level II or lower hospitals. For the definition of economically developed and underdeveloped areas in China, please refer to Multimedia Appendix 3. Data on the adoption of EHRs in US hospitals from 2008 to 2017 were obtained from the ONC data brief [2,3,16] and research by Jha et al [1,23-31], in which large hospitals were defined as those with ≥400 beds, while small and medium hospitals were those with 6-399 beds. Jha et al did not publish the number of surveyed and respondent hospitals in various subcategories in 2011 [28] and 2013 [26]. Since the ONC changed its statistical method after 2015, it only published the overall EHR adoption rate of US hospitals but not the rates in various subcategories. Therefore, only the numbers of surveyed and respondent hospitals for 2016 and 2017 are included in Figure 1.

Detailed information about the scale of the Sino-American hospital (including the different hospital types) is provided in Multimedia Appendix 4.

#### Adoption of EHRs in Chinese and US Hospitals

**Figure 2:**
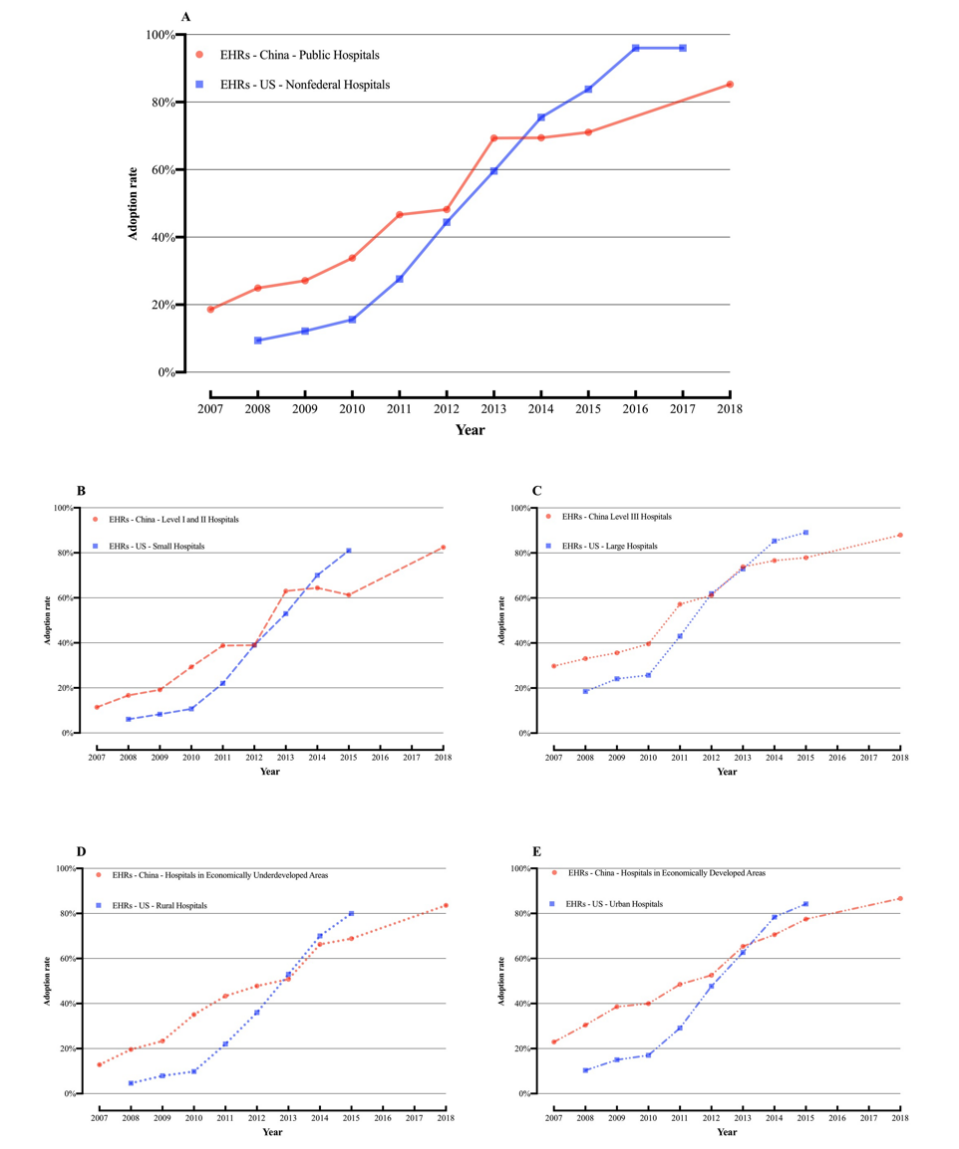
Trends in electronic health record (EHR) adoption rates in Chinese hospitals from 2007 to 2018 and nonfederal US hospitals from 2008 to 2017. (A) Overall adoption rate in China vs the United States and adoption rates in (B) small-scale hospitals, (C) large-scale hospitals, (D) hospitals in economically underdeveloped or rural areas, and (E) hospitals in economically developed or urban areas.

Trends in EHR adoption in China and the United States were compared (Figure 2), which revealed 3 main characteristics. First, the EHR adoption rates in China were relatively high. Overall, the average EHR adoption rates of the sampled Chinese hospitals in 2018 (85.3%) were 1.5% higher than those of US hospitals in 2015 (83.8%), but lower than those of US hospitals in 2017 (96%). To note here, since the ONC changed its statistical method after 2015, it published only the overall EHR adoption rate of US hospitals but not the rates in various subcategories. Therefore, only the data for 2016 and 2017 are included in Figure 2A. Considering hospital scale, the adoption rates in Level II or lower Chinese hospitals (small-scale hospitals) were 1.5% higher than in small hospitals (fewer than 100 beds) in the United States in 2015 (the adoption rate of the former being 82.5% compared to the 81% of the latter). However, the average adoption rate of Level III hospitals in China (87.9%) was 1.2% lower than that of large US hospitals (89.1%). Considering regional economic development, the average adoption rate in Chinese hospitals in economically underdeveloped regions was 3.6% higher than in rural US hospitals—83.6% and 80%, respectively. The adoption rate in economically developed Chinese hospitals (86.6%) was 2.4% higher than that in urban US hospitals (84.2%).

**Figure 3:**
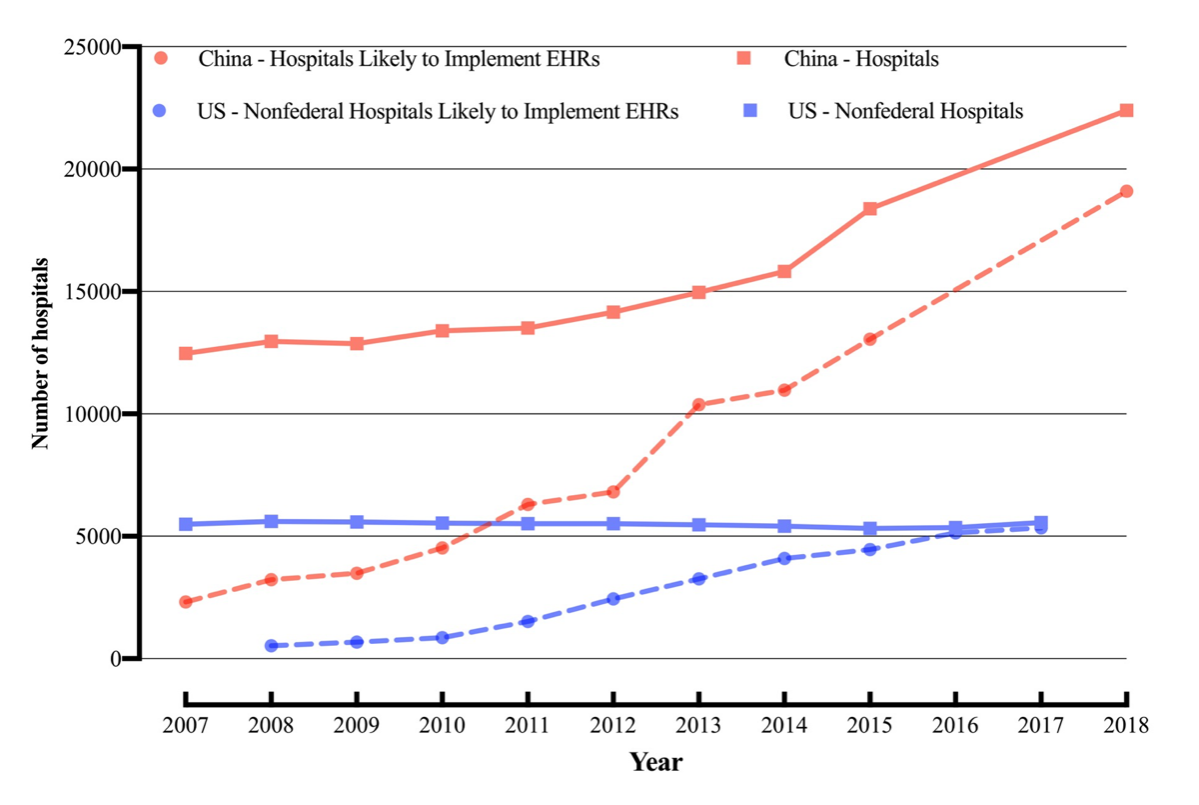
Numbers of Chinese hospitals and those likely to implement electronic health records (EHRs) from 2007 to 2018 and the numbers of nonfederal US hospitals and those projected to implement EHRs from 2008 to 2017.

Because the overall number of hospitals in China exceeds the number of urban hospitals in the United States, the absolute number and challenges of Chinese hospitals adopting EHRs should be greater. The annual average number of hospitals adopting EHRs in China far exceeded the US average—1500 and 534, respectively. In 2007, China had 12,477 Level I-III hospitals [44] and an annual EHR adoption rate of 18.6%. According to sample projection, only 2322 Chinese hospitals used EHRs. In 2018, China had 22,396 Level I-III hospitals using EHRs [45], with an EHR adoption rate of 85.3% and a total of 19,094 hospitals. Thus, 16,772 hospitals in China adopted EHRs from 2007 to 2018—3.3 times the number of nonfederal hospitals adopting EHRs in the United States from 2008 to 2017 (4814), according to the projections based on the total number of nonfederal US hospitals (see Figure 3) [46].

#### Difficulties With HIT Development in Chinese and US Hospitals

**Figure 4:**
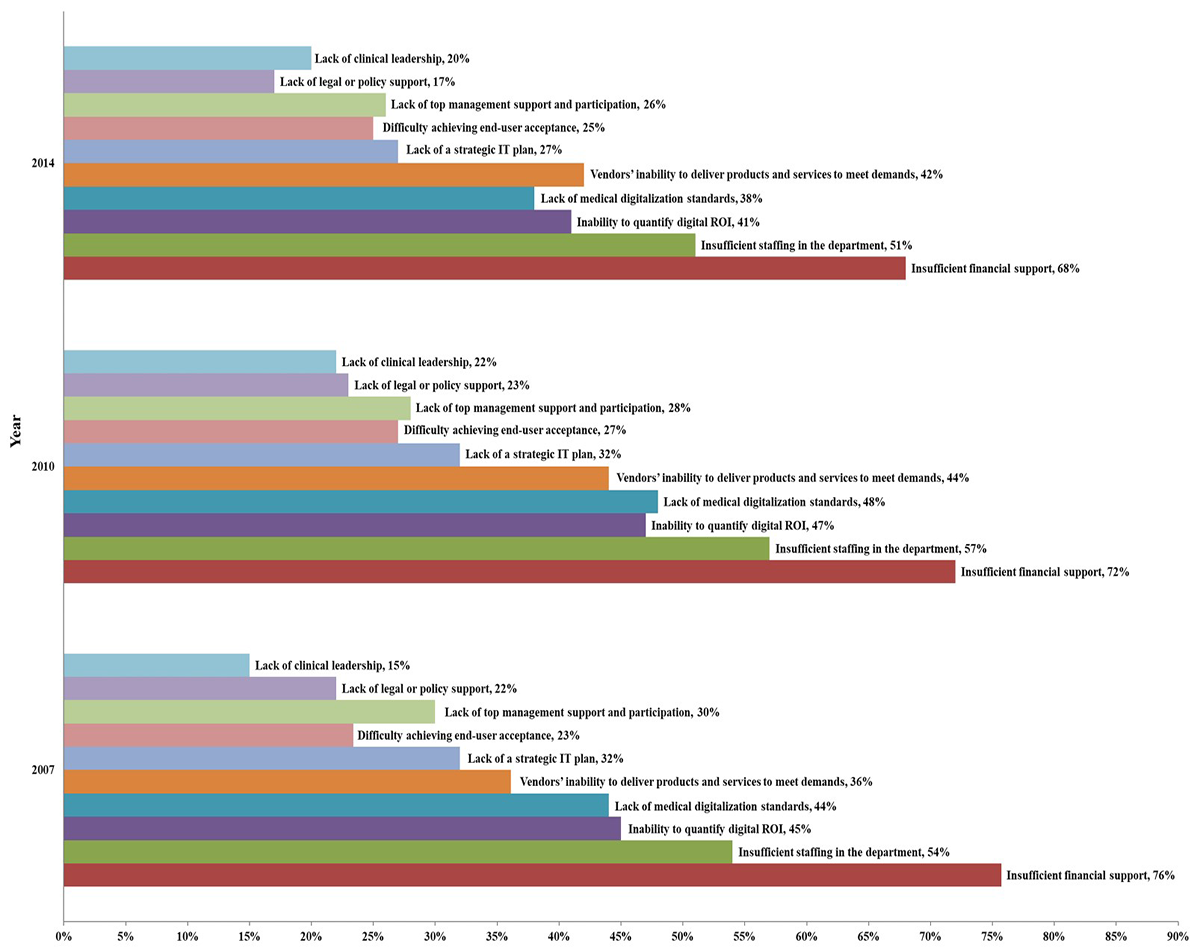
Survey feedback on health information technology (HIT) development barriers faced by hospitals in China in 2007, 2010, and 2014. IT: information technology; ROI: return on investment.

**Figure 5:**
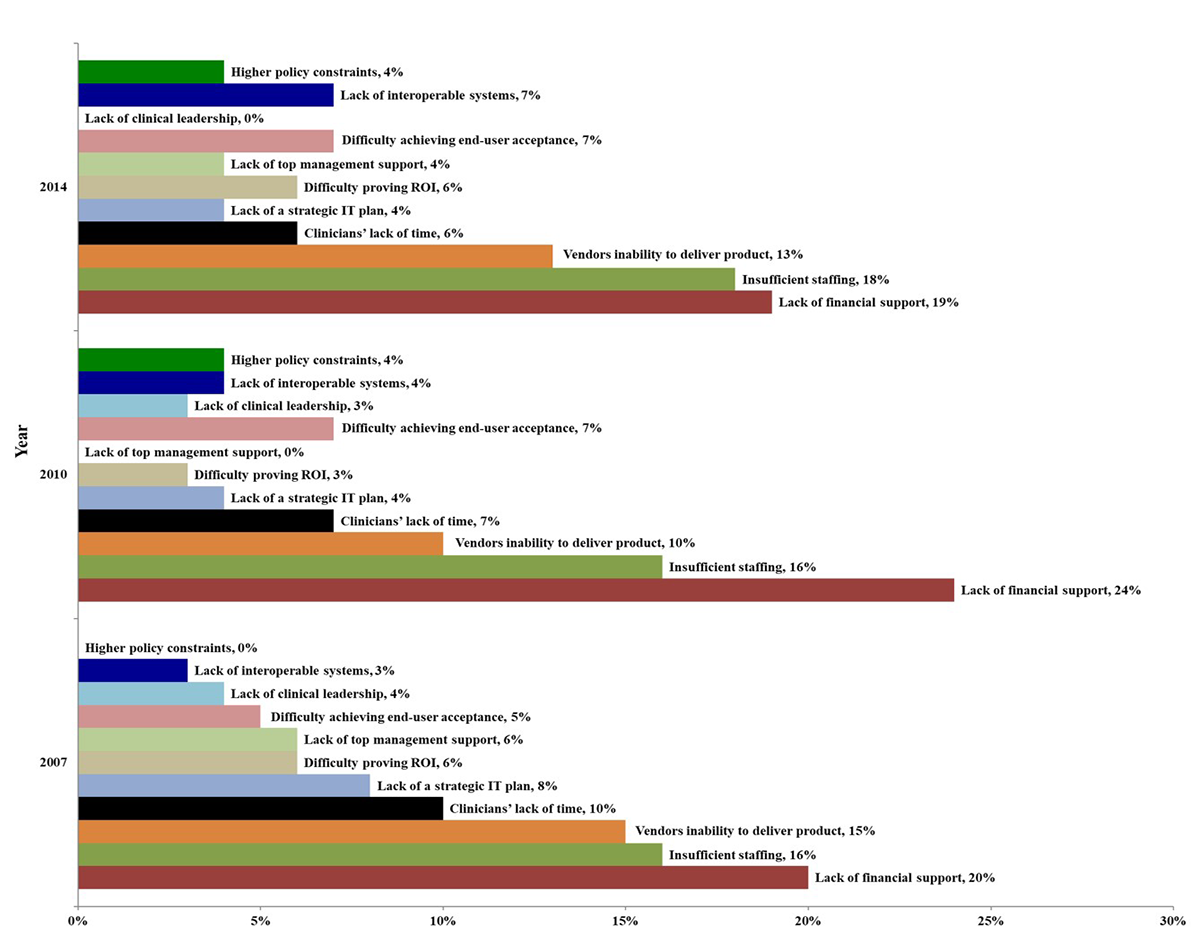
Survey feedback on health information technology (HIT) development barriers faced by hospitals in the United States in 2007, 2010, and 2014. IT: information technology; ROI: return on investment.

Figures 4 and 5 present the feedback from chief information officers (CIOs) on the barriers faced in the HIT application from the CHMIA and HIMSS surveys; as of 2015, the HIMSS Annual Survey no longer conducts a survey of hospital CIOs regarding the barriers to HIT application. Among Chinese hospitals, insufficient financial support and insufficient staff in the department were consistently identified as the first and second greatest obstacles, respectively. HIMSS Annual Survey data from the same years (2007, 2010, and 2014) show that US hospital CIOs also identified insufficient financial support and insufficient staff as their greatest challenges. This indicates a similarity in the main obstacles faced by China and the United States in hospital digitalization. In 2014, Chinese and US hospitals identified vendors’ inability to deliver products and services to meet their demands as the third greatest obstacle. This may be because, with the increasing development of HIT in hospitals, hospital CIOs have become increasingly demanding with regard to the relevant software products.

### Bass Model Fitting and Prediction of EHR Adoption Rates in Chinese and US Hospitals

Considerable differences between Chinese and US hospitals in terms of the EHR technology diffusion modes were identified. Using Bass modeling and linear optimization, we estimated *p* and *q* coefficients based on the CHIMA data from 2007 to 2018 [22] and the surveys from 2008 to 2017 reported by the ONC [2,3,16] and Jha et al [1,23-31]. The parameter estimation results of the final model (Table 1) indicated that the Bass model fit the CHIMA dataset [22] and the ONC [2,3,16] and Jha et al [1,23-31] datasets. The adjusted *R*^2^ was >0.9 for all models except the EHRs-China-Level III Hospitals model. Generally, each model shows a smaller motivation coefficient ratio (*q*/*p*) for Chinese hospitals compared to US hospitals. The largest difference (285-fold) was observed between the EHRs-China-Hospitals in the Economically Developed Areas model and the EHRs-US-Urban Hospitals model, which are the models of the largest-scale hospitals in these countries. In contrast, the smallest gap (14.8-fold) was observed between the EHRs-China-Surveyed Level I and II Hospitals model and the EHRs-US-Small Hospitals model, which are the models of these countries’ smallest-scale hospitals. Moreover, the internal *q* of US hospitals was significantly larger than that of Chinese hospitals. The largest difference (57-fold) was observed between the EHRs-China-Hospitals in the Economically Developed Areas model and the EHRs-US-Urban Hospitals model, while the smallest (3.6-fold) was found between the EHRs-China-Level I and II hospitals model and the EHRs-US-Small Hospitals model.

**Table 1:** Bass model parameters for the prevalence of electronic health records (EHRs) in Chinese and US hospitals, based on Chinese Health Information Management Association (CHIMA) data from 2007 to 2018 [22] and survey data reported by the Office of the National Coordinator for Health Information Technology (ONC) [2,3,16] and Jha et al [1,23-31] from 2008 to 2017.

Model	Model parameters			
	*p* ^a^	*q* ^b^	*q*/*p*^c^	Adjusted *R*^2^
EHRs-China	0.10	0.11	1.04	0.93
EHRs-China-Level I and II Hospitals	0.07	0.17	2.66	0.94
EHRs-China-Level III Hospitals	0.17	0.01	0.06	0.88
EHRs-China-Hospitals in Economically Underdeveloped Areas	0.08	0.13	1.51	0.98
EHRs-China-Hospitals in Economically Developed Areas	0.15	0.01	0.07	0.90
EHRs-US-Nonfederal Hospitals	0.02	0.58	24.33	0.97
EHRs-US-Small Hospitals	0.02	0.63	39.44	0.99
EHRs-US-Large Hospitals	0.07	0.45	6.64	0.95
EHRs-US-Rural Hospitals	0.01	0.65	46.29	0.99
EHRs-US-Urban Hospitals	0.03	0.57	19.66	0.98

^a^*p*: external motivation coefficient.

^b^*q*: internal motivation coefficient.

^c^*q*/*p*: motivation coefficient ratio.

**Figure 6:**
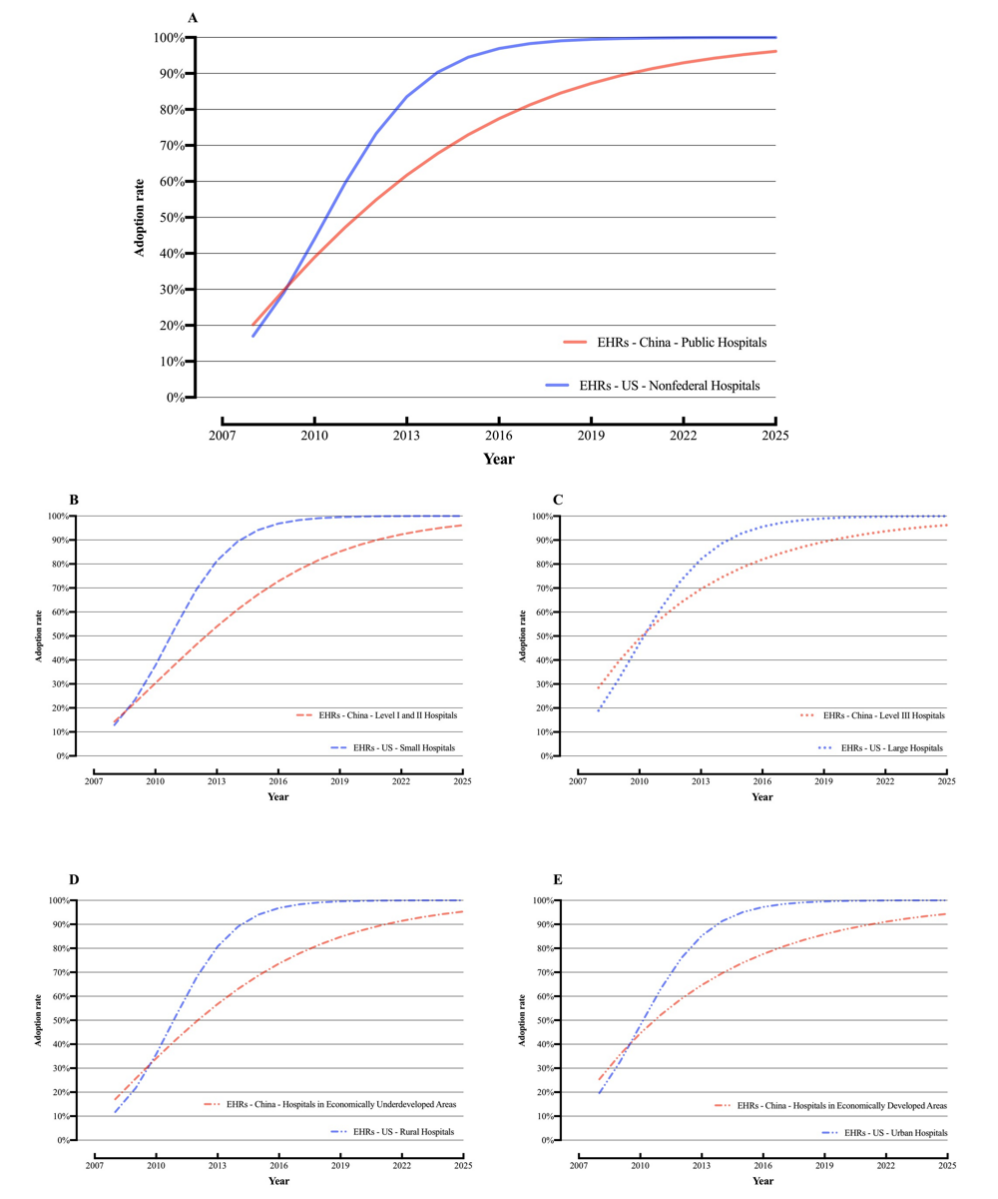
Predicted electronic health record (EHR) adoption up to 2025 in Chinese hospitals (based on Chinese Health Information Management Association Annual Survey data from 2007 to 2018) and in US hospitals (based on annual survey data from 2008 to 2017 reported by the Office of the National Coordinator for Health Information Technology and Jha et al [1,23-31]). (A) Overall predictions and predictions for (B) small-scale hospitals, (C) large-scale hospitals, (D) hospitals in economically underdeveloped or rural areas, and (E) hospitals in economically developed or urban areas.

The differing diffusion patterns of EHRs in Chinese and US hospitals led to the differences in the patterns of the diffusion dynamics curves. By assuming that there will be no major policy adjustments or technological advancements in the future, we used the Bass model to fit and predict future EHR adoption in Chinese and US hospitals from 2019 to 2025, both in the overall scale and according to hospital scale and location (Figure 6). An Annual Survey was not conducted in 2016 due to changes in CHIMA’s leadership. In 2017, the survey data from the software portion of the CHIMA Annual Survey deviated greatly; CHIMA does not recommend use of these data. Since the ONC changed its statistical method after 2015, it published only the overall EHR adoption rate of US hospitals but not the rates of various subcategories. Therefore, in Figures 6B, 6C, 6D, and 6E, the EHR adoption rates of various types of hospitals in the United States are predicted using the data from 2008 to 2015. The diffusion dynamics curve for EHRs in US hospitals forms a classic *S*-shape with a fast growth rate (*p*=0.03±0.02, *q*=0.58±0.07)—larger than that of Chinese hospitals (*p*=0.11±0.04, *q*=0.08±0.07).

## Discussion

Based on the 2007-2018 CHIMA Annual Surveys, we examined the progress and modes of EHR technology diffusion in sampled Chinese hospitals nationwide, identified major difficulties in HIT innovation, and compared them with US hospitals.

### Principal Findings

From the perspective of EHR implementation in Chinese hospitals, Chinese hospitals demonstrated differences in EHR adoption and growth rates according to scale and location. Among the sampled hospitals in China, the adoption rates in small hospitals (Level II or lower) and in hospitals from economically underdeveloped areas were below average. However, the growth rate of EHR adoption in these disadvantaged hospitals surpassed that of advantaged hospitals, as shown by the considerably higher slope in the Bass curves in Figure 6. This phenomenon is linked to national conditions, the medical system, and the financial support policy for HIT in hospitals in China. First, China has a vast territory that varies greatly from region to region. Although a large number of Level II or lower hospitals has been set up to provide basic medical services for local residents, high-quality medical resources are concentrated in a few Level III hospitals. Second, because China has not established a graded hierarchical medical system, patients are more inclined to congregate in Level III hospitals, leading to significantly higher workloads for doctors and correspondingly higher economic benefits [47,48]. As a result, many hospitals that are smaller or located in underdeveloped areas lack funds, resources, and motivation to build and maintain EHRs. Fortunately, the government has recognized this problem. Policies and funds should favor Level II or lower hospitals or those in underdeveloped areas, whereas Level III hospitals or those in developed areas should mostly be guided by policies and required to generate their own funding. Furthermore, the allowing of disadvantaged hospitals that have implemented EHRs to join medical institution alliances based on regional HIT has retained more patients in local hospitals instead of them seeking care in Level III hospitals, which also works to increase disadvantaged hospitals’ income [11]. With the rapid development and wide application of wearable device technology [49,50], more real-time health data can be included in EHRs, which will further promote this trend.

From the perspective of the comparison of the EHR adoption by Chinese hospitals and US hospitals, although both the Chinese and US governments have implemented policy guidelines and financial incentives to promote EHR adoption, the patterns of EHR diffusion between the 2 countries differ considerably. The graph shapes in Figure 2 show this difference. The US trend is more *S*-shaped and more typical of a market-driven diffusion pattern, while the Chinese trend is more linear and more like a top-down, policy-driven pattern. EHR adoption in Chinese hospitals follows the innovator mode (motion coefficient ratio *q/p* is only 0.06 to 2.66), indicating that hospitals began to use EHRs in the initial stages due to the influence of external administrative forces [51]. This is because, in China, most (about 71.1%) of the secondary and higher hospitals are funded and managed by the government, and the number of beds in public hospitals is 3 times higher (about 76%) than that of private hospitals. In 2010, the government began to invest considerable resources and funds into EHRs and issued relevant policies to guide and support their use. However, the HIT support strategy at the time did not provide a detailed, clear, and measurable meaning of EHRs in Chinese hospitals nor establish any quantitative rewards, penalties, or standards for the use of EHRs by hospitals. This led to weak growth after the initial implementation of financial support. Reliance on hospital motivation to promote EHRs without sufficient external financial and policy incentives was proven to be unrealistic and unsuccessful [4,5,52,53]. In contrast, the EHR adoption rate in US hospitals grew very slowly from 2008 to 2010, perhaps due to the adoption of the US Health Insurance Portability and Accountability Act [54] in 1996—a comprehensive personal electronic health information privacy and security protection law—and the fact that most (about 80%) of the hospitals are private, which should be promoted by economic interests. Since 2010, following the implementation of meaningful use programs with clear quantitative requirements for EHRs, the EHR adoption rate increased significantly, and most US hospitals started to use certified EHRs by 2017. In sharp contrast to the low effectiveness of the expansive HIT development strategy in China, the HITECH Act was a major driving force behind this progress [55,56]. The US government provides financial incentives to US hospitals that implement EHRs and meet meaningful use phased standards and imposes financial penalties on those that do not [30]. We believe that the financial support and policy guidance of this “carrot and stick” model is also one of the most important American experiences.

The most significant morphological difference between the hospitals’ EHR diffusion curves of China and the United States is that the motion coefficient ratio *q/p* value of the US curve is much larger. On one hand, the *q* value of the Chinese curve is smaller, and the *P* value is larger. This may be because the rapid spread of EHRs in China is caused by external policy stimuli. The Chinese government takes the HIT system represented by EHRs widely implemented in hospitals as a kind of technological innovation guided by the government and considers HIT as a technical tool to promote regional medical consortium [57]. As of 2015, according to our previous research results based on the same survey data, about 57.2% of the investigated hospitals have joined the regional medical consortium, 81.9% of these hospitals that have joined the medical consortium support the interconnection of electronic data, and the gap between HIT systems of different levels of medical institutions in the medical consortium is gradually narrowing [11]. On the other hand, the relatively large *q* value of the US curve may be interpreted in relation to 2 aspects. First, imitating the words or power of peers or industry leaders may influence American doctors to use similar EHRs as a tool for recording and exchanging health information [58]. Second, American doctors may have a strong willingness to upgrade information technology [59].

The comparison of the effects and outcomes of EHR implementation shows that the Chinese government has done more work to improve the implementation quantity and quality, as well as the relevant strategies used; has made unique contributions; and, thus, has had more achievements. This comparison can be made from 3 perspectives.

First, as for the implementation quantity, the EHR implementation rate in China in 2018 (85.3%) is equivalent to that in the United States in 2015 (83.8%) but is lower than that in the United States in 2017 (96%). However, since the base number of the former surpasses the latter (number of Chinese hospitals in 2018 was 22,396, compared to the number of nonfederal US hospitals in 2017, which was 5564) [46], the number of hospitals adopting EHRs in Chinese hospitals is approximately 3.3 times that in the United States—16,772 and 4818, respectively. Moreover, the annual growth of the former (1500) is about 2.8 times that of the latter (534). As of 2018, although China’s population (1.4 billion) is 4.28 times that of the United States (327 million), given that the Chinese gross domestic product (GDP) is only 67.8% that of the latter (the Chinese GDP being US $13.89 trillion, compared to the US $20.5 trillion GDP in the United States), the per capita GDP of China (US $9900) is only 15.8% that of the latter (US $62,500) and is below the world average (US $11,300). It also reflects that the former has made great progress in promoting EHRs in a short time (11 years), under the promotion of a huge subjective initiative.

Second, as for the implementation quality, the China Health Commission has conducted many top-level design policies. First, the connotation of EHRs is clearly defined, and EHR adoption is divided into Levels 0 to 8. Second, through administrative instructions, different deadlines are set for hospitals at different levels. For example, by the end of 2020, all Level III and Level II hospitals must use at least Level IV and Level III EHRs, respectively. Namely, by the end of 2020 [60], 11,565 secondary and tertiary hospitals in China, accounting for 52% of the country’s 22,000 hospitals, must use at least Chinese stage 3 EHRs (roughly corresponding to basic EHRs with notes). This is 2.4 times the number of the 4818 nonfederal hospitals implementing EHRs in the United States in 2017 [2,16]. As of July 2020, 128 Chinese hospitals were tested and verified using EHRs that met the high-level (stages 5-7) standards—44 more than the same period of last year (84 hospitals)—of which 4 reached stage 7 (an increase of 2 hospitals) and 20 reached stage 6 (an increase of 15 hospitals) [61]. Moreover, the performance monitoring data of the Chinese government for public hospitals partially verify and support the prediction results of the BASS model. As of July 2020, the announcement on “the National Monitoring and Analysis of the Performance Appraisal of the National Tertiary Public Hospitals” released by the Chinese government in 2018 [62] shows that the participation rate of China’s EMR level evaluation of tertiary hospitals was 94.58% by 2018, with an average stage of 2.72—a stage close to the level of basic EHR with clinical notes in the United States. Approximately 87% of tertiary hospitals reached EHR Level III or above, which is very close to the prediction result of the Bass model (87.2%). We believe that as of the end of 2020, China’s tertiary hospitals were likely to achieve stage 4 EMRs (namely, comprehensive EHRs). The Chinese government has also released information, which was published near the end of 2020, on the results of the performance appraisal of hospitals, including the progress of the implementation of EMRs in public hospitals below the third level.

Third, as for the implementation strategy, evaluation is emphasized with the principle “promote construction with evaluation, promote improvement with evaluation*.*” The Chinese government has adopted different direct capital investments and indirect policy guidance strategies for hospitals of different scales. For the Level III large hospitals, which are responsible for over 46% of outpatients in China, the government focused on policy guidance, released many guidelines [63] and management and normative documents from 2010 to 2019 to promote hospital digitization with EHRs as the core, and encouraged private capital investments [64]. Additionally, for small hospitals at a level lower than Level III, a strategy of direct finance and indirect guidance was adopted to gradually promote EHR implementation. Furthermore, in 2019, the State Council of China stipulated that the construction of EHRs was one major indicator for hospital-level assessment and appointment of public hospital presidents [64]. For example, in the 3-year national hospital evaluation, the EHRs used by tertiary hospitals must meet Chinese stage 4; otherwise, the hospitals will be downgraded, which will greatly affect the reputation and economic income of the hospitals.

### Limitations

The data used here were collected from (1) repeated measurements of EHR constructions in the same batch of US hospitals affiliated with the ONC and American Hospital Association (2007-2017) and (2) repeated investigations through self-report questionnaires (2007-2015, 2018) of EHR construction from Chinese hospitals participating in annual conferences organized by the CHIMA. The latter was not independently verified. Therefore, such analysis might be affected by several potential confounding factors of data bias. First, due to the limitation of the CHIMA survey data, there may be limitations to the classification of EHRs in hospitals in China. However, the implementation rate of each classification of hospitals is only an added reference index. Moreover, there are no such hospitals in the United States, so this classification is not used mainly for the comparison of the same hospitals in China and the United States. Second, we did not use multivariate models to assess the independence among different factors (eg, grades, types, economic levels, or locations of hospitals). Third, the cumulative proportions of some repeated questionnaire data from CHIMA during 2007-2015 slightly declined. We think one explanation may be that throughout the repeated surveys, the sampling differences of hospital samples led to differences in the investigated data. Although we limit our deductions to our own samples, our analyses are valuable in that these data are the only available quantitative data concerning the trend in HIT development in China over a time span of 10 years and collected by the Chinese state-level academy in this field. These are the only authoritative, national-level, long-term quantitative data available on the rate of adoption of EHRs in Chinese hospitals. Therefore, these are the best available data that can reflect the status of EHR use in Chinese hospitals. Furthermore, due to the differences in the economic, cultural, and health systems between China and the United States, there are also some differences in the functional definition of necessary components of hospital EHRs. Therefore, we mainly analyzed the overall time trend of EHR implementation in hospitals of the 2 countries, and the horizontal comparison is only for an approximate reference.

### Conclusion

Over the last decade, the Chinese government has identified HIT development, represented by hospital EHRs, as an important technical focus and starting point to support medical reform. According to the CHIMA Annual Surveys, the average EHR adoption rate in sampled hospitals in China increased by 3.6 times from 2007 to 2018, peaking at 85.3%, which exceeds that of 83.8% in US hospitals in 2015 but is lower than the 96% recorded in 2017. The difference in the EHR technology diffusion curves of China and the United States based on the Bass model is very likely due to the differences in the EHR promotion, implementation, and management policies, as well as the medical system, of the 2 countries. The former is mainly stimulated by external policies, while the latter is initiated by their own technological upgrading needs. The Chinese government has begun to amend relevant policies, gradually implementing both financial support and policy guidance measures and adding the assessment of secondary utilization based on precipitated data on EHRs and the use of various advanced functions. This action technically underlies several medical reform goals, such as improving clinical outcomes, user satisfaction, and interoperability. Various signs indicate that the Chinese government is gradually approaching and realizing its phase goals established in the second medical reform initiated in 2010, including the integration of medical resources, improvement of the popularization and quality of medical care, and the reduction of medical costs.

## Appendix

Multimedia Appendix 1 Electronic health record (EHR) functions used to define "basic without clinical notes," "basic with clinical notes," and "comprehensive" EHR systems.

Multimedia Appendix 2 Chinese evaluation and management of application level of EHRs system.

Multimedia Appendix 3 The definitions of economically developed and underdeveloped areas in China from CHIMA Annual Surveys of Hospital Information Systems.

Multimedia Appendix 4 Scale of the 2007-2018 CHIMA Annual Surveys of Hospital Information Systems and the 2008-2017 surveys on adoption of EHRs in U.S. hospitals.

## Abbreviations

CHIMA: Chinese Hospital Information Management Association
CIO: chief information officer
CMS: Centers for Medicare & Medicaid Services
EHR: electronic health record
EMR: electronic medical record
EMRAM: EMR adoption model
GDP: gross domestic product
HIT: health information technology
HITECH: Health Information Technology for Economic and Clinical Health
ONC: Office of the National Coordinator for Health Information Technology

## References

[1] Jha AK, Ferris TG, Donelan K, DesRoches C, Shields A, Rosenbaum S, Blumenthal D (2006). How common are electronic health records in the United States? A summary of the evidence. Health Aff (Millwood).

[2] Henry J, Pylypchuk Y, Searcy T, Patel V (2016). Adoption of Electronic Health Record Systems among U.S. Non-Federal Acute Care Hospitals: 2008-2015. Office of the National Coordinator for Health Information Technology.

[3] (2019). Non-federal Acute Care Hospital Health IT Adoption and Use. Office of the National Coordinator for Health Information Technology.

[4] Kim Y, Jung K, Park Y, Shin D, Cho S, Yoon D, Park RW (2017). Rate of electronic health record adoption in South Korea: A nation-wide survey. Int J Med Inform.

[5] Kanakubo T, Kharrazi H (2019). Comparing the Trends of Electronic Health Record Adoption Among Hospitals of the United States and Japan. J Med Syst.

[6] Esdar M, Hüsers J, Weiß JP, Rauch J, Hübner U (2019). Diffusion dynamics of electronic health records: A longitudinal observational study comparing data from hospitals in Germany and the United States. Int J Med Inform.

[7] (2015). Technical specification of hospital information platform based on electronic medical record. National Health Commission of the People's Republic of China.

[8] Garets D, Davis M (2006). Electronic medical records vs electronic health records: yes, there is a difference. American Academy of Ophthalmology.

[9] Gold M, McLaughlin C (2016). Assessing HITECH Implementation and Lessons: 5 Years Later. Milbank Q.

[10] Colicchio TK, Cimino JJ, Del Fiol G (2019). Unintended Consequences of Nationwide Electronic Health Record Adoption: Challenges and Opportunities in the Post-Meaningful Use Era. J Med Internet Res.

[11] Liang J, Zheng X, Chen Z, Dai S, Xu J, Ye H, Zhang Z, Ge F, Brand J (2019). The experience and challenges of healthcare-reform-driven medical consortia and Regional Health Information Technologies in China: A longitudinal study. Int J Med Inform.

[12] (2018). Electronic Medical Record System Application Level Grading Evaluation Standard (Trial). National Health Commission of the People's Republic of China.

[13] (2019). Hospital Smart Service Rating Evaluation Standard System (Trial). National Health Commission of the People's Republic of China.

[14] Liang J, Li Y, Zhang Z, Shen D, Xu J, Yu G, Dai S, Ge F, Lei J (2020). Evaluating the Applications of Health Information Technologies in China During the Past 11 Years: Consecutive Survey Data Analysis. JMIR Med Inform.

[15] Feng Z (2018). Health Care For 1.4 Billion: China’s Healthcare System And Reform. Health Affairs.

[16] Charles D, Gabriel M, Searcy T (2015). Adoption of Electronic Health Record Systems among U.S. Non-Federal Acute Care Hospitals: 2008-2014. Office of the National Coordinator for Health Information Technology.

[17] (2013). What is meaningful use?. The Office of the National Coordinator for Health Information Technology.

[18] (2015). Electronic Medical Record Adoption Model. HIMSS Analytics.

[19] (2018). Notice on Further Promoting the digitalization Construction of Medical Institutions with Electronic Medical Records as the Core. National Health Commission of the People's Republic of China.

[20] Xu L, Wen D, Zhang X, Lei J (2016). Assessing and comparing the usability of Chinese EHRs used in two Peking University hospitals to EHRs used in the US: A method of RUA. Int J Med Inform.

[21] Healthcare Information and Management Systems Society (2019). Public hospitals are not allowed to participate in HIMSS ratings. MedSci/Policy and Humanities.

[22] Luo S, Zhang K, Li B (2010). Medical informatics in China: healthcare IT trends, academic and research developments. Yearb Med Inform.

[23] Sheikh A, Jha A, Cresswell K, Greaves F, Bates DW (2014). Adoption of electronic health records in UK hospitals: lessons from the USA. The Lancet.

[24] Furukawa MF, King J, Patel V, Hsiao C, Adler-Milstein J, Jha AK (2014). Despite substantial progress In EHR adoption, health information exchange and patient engagement remain low in office settings. Health Aff (Millwood).

[25] Adler-Milstein J, DesRoches CM, Kralovec P, Foster G, Worzala C, Charles D, Searcy T, Jha AK (2015). Electronic Health Record Adoption In US Hospitals: Progress Continues, But Challenges Persist. Health Aff (Millwood).

[26] Adler-Milstein J, DesRoches CM, Furukawa MF, Worzala C, Charles D, Kralovec P, Stalley S, Jha AK (2014). More than half of US hospitals have at least a basic EHR, but stage 2 criteria remain challenging for most. Health Aff (Millwood).

[27] Ford EW, Menachemi N, Peterson LT, Huerta TR (2009). Resistance is futile: but it is slowing the pace of EHR adoption nonetheless. J Am Med Inform Assoc.

[28] DesRoches CM, Worzala C, Joshi MS, Kralovec PD, Jha AK (2012). Small, nonteaching, and rural hospitals continue to be slow in adopting electronic health record systems. Health Aff (Millwood).

[29] Jha AK, DesRoches CM, Campbell EG, Donelan K, Rao SR, Ferris TG, Shields A, Rosenbaum S, Blumenthal D (2009). Use of electronic health records in U.S. hospitals. N Engl J Med.

[30] Adler-Milstein J, Holmgren AJ, Kralovec P, Worzala C, Searcy T, Patel V (2017). Electronic health record adoption in US hospitals: the emergence of a digital "advanced use" divide. J Am Med Inform Assoc.

[31] Jha AK, Burke MF, DesRoches C, Joshi MS, Kralovec PD, Campbell EG, Buntin MB (2011). Progress toward meaningful use: hospitals' adoption of electronic health records. Am J Manag Care.

[32] Healthcare Information and Management Systems Society (2006). 17th Annual 2006 HIMSS Leadership Survey, Healthcare CIO Final Report. HIMSS February 13. Report No.

[33] Healthcare Information and Management Systems Society (2007). 18th Annual 2007 HIMSS Leadership Survey, Healthcare CIO Final Report. HIMSS April 10. Report No.

[34] Healthcare Information and Management Systems Society (2010). 21th Annual 2010 HIMSS Leadership Survey, Healthcare CIO Final Report. HIMSS February 19. Report No.

[35] Healthcare Information and Management Systems Society (2014). 25th Annual 2014 HIMSS Leadership Survey Healthcare CIO Final Report. HIMSS February 24. Report No.

[36] Bass FM (2004). A New Product Growth for Model Consumer Durables. Management Science.

[37] Van de Bulte C (2002). Want to know how diffusion speed varies across countries and products? Try using a Bass model. PDMA visions.

[38] Sood A, James GM, Tellis GJ, Zhu J (2012). Predicting the Path of Technological Innovation: SAW vs. Moore, Bass, Gompertz, and Kryder. Marketing Science.

[39] Kharrazi H, Gonzalez CP, Lowe KB, Huerta TR, Ford EW (2018). Forecasting the Maturation of Electronic Health Record Functions Among US Hospitals: Retrospective Analysis and Predictive Model. J Med Internet Res.

[40] Ford EW, Hesse BW, Huerta TR (2016). Personal Health Record Use in the United States: Forecasting Future Adoption Levels. J Med Internet Res.

[41] Norton JA, Bass FM (1987). A Diffusion Theory Model of Adoption and Substitution for Successive Generations of High-Technology Products. Management Science.

[42] Grimm SE, Stevens JW, Dixon S (2018). Estimating Future Health Technology Diffusion Using Expert Beliefs Calibrated to an Established Diffusion Model. Value Health.

[43] Lei J, Guan P, Gao K, Lu X, Chen Y, Li Y, Meng Q, Zhang J, Sittig DF, Zheng K (2014). Characteristics of health IT outage and suggested risk management strategies: an analysis of historical incident reports in China. Int J Med Inform.

[44] Chen Z, National Health Commission of the People's Republic of China (2010). 2010 China Health Statistics Yearbook.

[45] Ma X, National Health Commission of the People's Republic of China (2019). 2019 China Health Statistics Yearbook.

[46] Elflein J (2020). Number of federal and non-feaderal hospitals in the US from 1957 to 2017. Statista.

[47] Fu Y, Schwebel D, Hu G (2018). Physicians' Workloads in China: 1998⁻2016. Int J Environ Res Public Health.

[48] Wen J, Cheng Y, Hu X, Yuan P, Hao T, Shi Y (2016). Workload, burnout, and medical mistakes among physicians in China: A cross-sectional study. Biosci Trends.

[49] Wen D, Zhang X, Liu X, Lei J (2017). Evaluating the Consistency of Current Mainstream Wearable Devices in Health Monitoring: A Comparison Under Free-Living Conditions. J Med Internet Res.

[50] Xie J, Wen D, Liang L, Jia Y, Gao L, Lei J (2018). Evaluating the Validity of Current Mainstream Wearable Devices in Fitness Tracking Under Various Physical Activities: Comparative Study. JMIR Mhealth Uhealth.

[51] Rogers EM (2003). Diffusion of Innovations, 5th Edition.

[52] Yoon D, Chang B, Kang S, Bae H, Park R (2012). Adoption of electronic health records in Korean tertiary teaching and general hospitals. Int J Med Inform.

[53] Yoshida Y, Imai T, Ohe K (2013). The trends in EMR and CPOE adoption in Japan under the national strategy. Int J Med Inform.

[54] Bendix J (2013). HIPAA: how to protect yourself and your practice. Med Econ.

[55] Cohen MF (2016). Impact of the HITECH financial incentives on EHR adoption in small, physician-owned practices. Int J Med Inform.

[56] Mennemeyer ST, Menachemi N, Rahurkar S, Ford EW (2016). Impact of the HITECH Act on physicians' adoption of electronic health records. J Am Med Inform Assoc.

[57] Wang Z (2019). Data integration of electronic medical record under administrative decentralization of medical insurance and healthcare in China: a case study. Isr J Health Policy Res.

[58] Yaraghi N, Du AY, Sharman R, Gopal RD, Ramesh R, Singh R, Singh G (2014). Professional and geographical network effects on healthcare information exchange growth: does proximity really matter?. J Am Med Inform Assoc.

[59] Whitney E (2015). Sharing Patient Records Is Still A Digital Dilemma For Doctors. Morning Edition.

[60] (2018). Notice on the issuance of the administrative measures for grading evaluation of the application level of electronic medical records system (trial implementation) and evaluation standards (trial implementation). National Health Commission of the People's Republic of China.

[61] (2020). Full analysis of 128 high-level evaluation hospitals with electronic medical records. CN-Healthcare.

[62] (2020). Announcement on the National Monitoring and Analysis of the Performance Appraisal of the National Tertiary Public Hospitals in 2018. National Health Commission of the People's Republic of China.

[63] Fu Y, Schwebel D, Hu G (2018). Physicians' Workloads in China: 1998⁻2016. Int J Environ Res Public Health.

[64] Ting S, Haiyi L, Wei Z (2019). Enlightenment from the Development of Chinese and American Electronic Medical Record System in the Past Decade. China Digital Medicine.

