# Supplementary material for: Adoption of Electronic Health Records (EHRs) in China During the Past 10 Years: Consecutive Survey Data Analysis and Comparison of Sino-American Challenges and Experiences
Source: J Med Internet Res. 2021 Feb 18;23(2):e24813. doi: 10.2196/24813 (PMC7932845; doi:10.2196/24813)
Supplement: Multimedia Appendix 1 [file jmir_v23i2e24813_app1.docx]

Appendix 1 EHR functions used to define ‘basic without clinical notes’, ‘basic with clinical notes’ and ‘comprehensive’ EHRs systems

| EHR Functions | Basic without clinical notes | Basic EHR with clinician notes | Comprehensive EHR |
| --- | --- | --- | --- |
| Patient Demographics | X | X | X |
| Physician notes |  | X | X |
| Nursing Assessments |  | X | X |
| Problem lists | X | X | X |
| Medication lists | X | X | X |
| Discharge summaries | X | X | X |
| Advance directives |  |  | X |
| Lab reports |  |  | X |
| Radiology tests |  |  | X |
| Medications | X | X | X |
| Consultation requests |  |  | X |
| Nursing orders |  |  | X |
| View lab reports | X | X | X |
| View radiology reports | X | X | X |
| View radiology images |  |  | X |
| View diagnostic test results | X | X | X |
| View diagnostic test images |  |  | X |
| View consultant report |  |  | X |
| Clinical guidelines |  |  | X |
| Clinical reminders |  |  | X |
| Drug allergy results |  |  | X |
| Drug-drug interactions |  |  | X |
| Drug-lab interactions |  |  | X |
| Drug dosing support |  |  | X |
