# Supplementary material for: Adoption of Electronic Health Records (EHRs) in China During the Past 10 Years: Consecutive Survey Data Analysis and Comparison of Sino-American Challenges and Experiences
Source: J Med Internet Res. 2021 Feb 18;23(2):e24813. doi: 10.2196/24813 (PMC7932845; doi:10.2196/24813)
Supplement: Multimedia Appendix 2 [file jmir_v23i2e24813_app2.docx]

Appendix 2 Chinese evaluation and management of application level of EHRs system

The Obama Administration signed ARRA in 2009, promising to invest USD 19 billion into MIT, and later announced the HITECH economic stimulus act, core of which was to promote "Meaningful Use" of electronic medical records. National Health Commission of China started the annotation of electronic medical record application levels since 2010, aiming to benchmark the MU act of the U.S., to comprehensively and objectively evaluate the current application levels of electronic medical records, to establish an evaluation system and an improved system suitable for China, and to execute these systems in 2018 after further improvements. The scheme integrated quantitative scoring and overall classification. The quantitative part was descriptive assessment based on data requirements and avoided as much as possible the subjective influence from self-assessment and expert assessment. Of the scores of levels 0-8 (totally 9 levels), levels 0-2 were low stage, levels 3-5 were medium stage, and levels 6-8 were high stage.

1. Level 0: Absence of electronic medical record system.
2. Level 1: Established independent medical information system.
3. Level 2: Exchange within the medical information department.
4. Level 3: Data exchange among departments. Medical care operating departments can exchange data on the Internet, and adopt any means (e.g. interface integration, data transfer from information systems) to acquire digitized data from outside of the department. Data of a certain department can be shared by other departments. Information systems have the checking and examining functions according to basic dictionaries. Data can be shared between at least two types of medical information departments (e.g. medical advices, examinations, tests, nursing). There is a unified trans-department medical care data dictionary.
5. Level 4: Information sharing across the whole hospital, and primary medical care decision support. Data exchange among all systems (e.g. HIS, LIS) is realized via data interfaces. EHRs have the ability to provide at least 1 examination function based on basic dictionaries and related with system data. Able to share the information of seeking medical assistance (including drug use, examination, tests, nursing, treatment, surgery) safely within the whole hospital. Automatic checking of drug prescription and interaction, and monitoring of rational drug use can be realized.
6. Level 5: Unified data management and medium-level medical care decision support. Different departments can utilize the integrated information and knowledge base unified within the whole hospital, provide a unified knowledge base involving clinical diagnosis and treatment standards, rational drug use, and clinical pathways, and offer integrated display and decision support to the department. Data from all systems of the hospital can be integrated according to a unified medical care data management mechanism, and trans-department integrated display tools can be offered. Able to support complete data acquisition and intellectualization tools are available, and structured and intelligent writing of medical records and reports. Based on integrated patient information, able to achieve the decision support services with the use of knowledge bases, and to offer data mining functions for medical management and clinical research.
7. Level 6: Full-course medical data closed-loop management, and high-level medical decision support. The functions of data acquisition, recording and sharing can be used by all medical care operational projects. Able to display the state throughout the course. Able to provide real-time data checking, display and control functions at the current step according to knowledge bases. Capable of full-course data tracking and closed-loop management, including checking, testing, therapy, surgery, blood transfusion, and nursing, and capable of full-course real-time data checking and management with the help of knowledge bases. Able to form a whole-hospital multidimensional medical knowledge base system (including symptoms, syndromes, examinations, tests, diagnosis, therapy, reasonable drug use and other relevant medical contents at different stages), and to provide high-level medical decision support.
8. Level 7: Medical care safety quality control, and regional medical information sharing. Able to comprehensively use medical information for medical safety and quality control throughout the whole department. Able to share medical information of patients outside the medical institution, and to conduct combined diagnosis and treatment. Able to collect medical quality and efficiency monitoring data from daily medication information systems, mainly including safe quality indices (within-hospital infection, adverse events, surgery), daily medical care operational efficiency indices, to have timely warning, notifying and reporting systems, and to offer intelligentized perception and analytical tools. Able to bidirectionally exchange patient information (including state of illness, examinations, tests, treatment) with external medical institutions. Able to ensure the problems of patient identification and information security have been solved during information exchange. Able to link diagnosis and treatment activities by using medical information both within and outside of the hospital. Patients can inquire their examination and test results and acquire information of drug use notification on the Internet.
9. Level 8: Integration of health information, and continual improvement of medical safety quality. Able to integrate across-institution information (including medical care, health records, symptom examinations and follow-up) into the medical activities of their own institution. Able to know the medical quality information related to their own department throughout the whole region, and uses it into the medical safety and quality continuous improvement in their own department. Able to comprehensively integrate information of medical care, public health and health monitoring, and to integrate medical services. Able to comparatively use regional medical quality indices, persistently monitor and manage the medical safety and quality levels of their own medical institution, and to continually improve.
