# Supplementary material for: Adoption of Electronic Health Records (EHRs) in China During the Past 10 Years: Consecutive Survey Data Analysis and Comparison of Sino-American Challenges and Experiences
Source: J Med Internet Res. 2021 Feb 18;23(2):e24813. doi: 10.2196/24813 (PMC7932845; doi:10.2196/24813)
Supplement: Multimedia Appendix 3 [file jmir_v23i2e24813_app3.docx]

Appendix 3 The *definitions of economically developed and underdeveloped areas in China* from CHIMA Annual Surveys of Hospital Information Systems

1. 2006: According to the per capita GDP in the previous year of the investigated area, per capita ≥ GDP¥15,000, ¥14,999-¥10,000, and < ¥10,000 were classified as economically developed area, economically medium-developed area, and economically underdeveloped area respectively.
2. 2007: According to the per capita GDP in the previous year of the investigated area, per capita GDP > ¥20,000, ¥20,000-¥10,000, and < ¥10,000 were classified as economically developed area, economically medium-developed area, and economically underdeveloped area respectively.
3. 2008: According to the per capita GDP in the previous year of the investigated area, per capita GDP > ¥30,000, ¥ 30,000-¥ 14,999, and ≤ ¥ 14,999 were classified as economically developed area, economically medium-developed area, and economically underdeveloped area respectively.
4. 2009: According to the per capita GDP in the previous year of the investigated area, per capita GDP > ¥35,000, ¥ 35,000-¥ 19,999, and ≤ ¥ 19,999 were classified as economically developed area, economically medium-developed area, and economically underdeveloped area respectively.
5. 2010, 2011: According to the per capita GDP in the previous year of the investigated area, per capita GDP > ¥ 40,000, ¥ 40,000-¥ 2,1000, and < ¥2, 1000 were classified as economically developed area, economically medium-developed area, and economically underdeveloped area respectively.
6. 2012: According to the per capita GDP in the previous year of the investigated area, per capita GDP >¥ 50,000, ¥ 50,000-¥ 30,000, and < ¥ 30,000 were classified as economically developed area, economically medium-developed area, and economically underdeveloped area respectively.
7. 2013: According to the per capita GDP in the previous year of the investigated area, per capita GDP >¥ 60,000, ¥ 60,000-¥ 30,000, and < ¥ 30,000 were classified as economically developed area, economically medium-developed area, and economically underdeveloped area respectively.

2014, 2015, 2017, 2018: According to the per capita GDP in the previous year of the investigated area, per capita GDP> ¥ 60,000, ¥ 60,000-¥ 35,000, and < ¥ 35,000 were classified as economically developed area, economically medium-developed area, and economically underdeveloped area respectively.
