# Supplementary material for: Adoption of Electronic Health Records (EHRs) in China During the Past 10 Years: Consecutive Survey Data Analysis and Comparison of Sino-American Challenges and Experiences
Source: J Med Internet Res. 2021 Feb 18;23(2):e24813. doi: 10.2196/24813 (PMC7932845; doi:10.2196/24813)
Supplement: Multimedia Appendix 4 [file jmir_v23i2e24813_app4.docx]

Appendix 4 Scale of the 2007-2018 CHIMA Annual Surveys of Hospital Information Systems and the 2008-2017 surveys on adoption of EHRs in U.S. hospitals.

|  | 2007 | 2008 | 2009 | 2010 | 2011 | 2012 | 2013 | 2014 | 2015 | 2017 | 2018 |
| --- | --- | --- | --- | --- | --- | --- | --- | --- | --- | --- | --- |
| China - Number of surveyed hospitals | 1161 | 1300 | 1028 | 1305 | 1004 | 1067 | 590 | 570 | 536 | 484 | 1909 |
| China - Number of surveyed level III hospitals | 448 | 629 | 474 | 656 | 512 | 371 | 344 | 335 | 448 | 263 | 974 |
| China - Number of surveyed level I and II hospitals | 713 | 671 | 554 | 649 | 492 | 696 | 246 | 235 | 194 | 221 | 935 |
| China - Number of surveyed hospitals in economically developed areas | 297 | 261 | 290 | 467 | 406 | 452 | 228 | 182 | 309 | 198 | 691 |
| China - Number of surveyed hospitals in economically underdeveloped areas | 864 | 1039 | 738 | 838 | 598 | 615 | 362 | 388 | 227 | 286 | 1218 |
| U.S - Number of surveyed and responded hospitals | / | 2952 | 3101 | 2902 | 2646 | 2796 | 2674 | 2640 | 2803 | 3304 | 3599 |
| U.S - Number of surveyed and responded large hospitals | / | 295 | 341 | 345 | * | 339 | * | 346 | 372 | + | + |
| U.S - Number of surveyed and responded small and medium hospitals | / | 2657 | 2760 | 2557 | * | 2457 | * | 2294 | 2431 | + | + |
| U.S - Number of surveyed and responded hospitals in urban | / | 1830 | 1675 | 2179 | * | 2104 | * | 1896 | 1694 | + | + |
| U.S - Number of surveyed and responded hospitals in nonurban | / | 1122 | 1426 | 723 | * | 692 | * | 744 | 1109 | + | + |
